# Biogenic amorphous calcium phosphate: a sustainable alternative for dentin remineralization

**DOI:** 10.1186/s12903-025-06524-y

**Published:** 2025-07-11

**Authors:** M. Madhana Madhubala, G. S. Nayantara, R. Jayasree, Janis Locs, Sekar Mahalaxmi

**Affiliations:** 1 Department of Conservative Dentistry and Endodontics, SRM Dental College, SRM Institute of Science and Technology, Ramapuram, Bharathi Salai, Chennai, Tamil Nadu 600089 India; 2 10X Assistive Technology, IITM Research Park, Taramani, Chennai, Tamil Nadu 600070 India; 3 Faculty of Natural Science and Technology, Institute of Biomaterials and Bioengineering, Riga Technical University, Riga, Latvia

**Keywords:** Dentin remineralization, Amorphous calcium phosphate, Eggshell-derived calcium phosphate, Microhardness, Antibacterial

## Abstract

**Background:**

Dentin demineralization weakens tooth integrity, requiring effective remineralization approaches. Amorphous calcium phosphate (ACP) is commonly used to deliver calcium and phosphate ions for dentin repair. However, synthetic ACP (SACP) often exhibits instability and limited antibacterial properties. In contrast, biogenic ACP (BACP), derived from eggshell waste, presents a sustainable alternative with potential advantages.

**Objective:**

This study aimed to compare the remineralization potential of SACP and BACP by evaluating their ability to restore microhardness, tubular occlusion, and crystal deposit formation. Antibacterial efficacy against *S. mutans* and *L. acidophilus* was also assessed.

**Methods:**

BACP was synthesized from eggshell waste using thermal and chemical processes, while SACP was commercially obtained. Both materials were characterized using SEM, EDX, FTIR, and XRD, with biocompatibility verified via MTT assay. Dentin specimens (*n* = 36) were demineralized (pH 4.5, 72 h) and divided into BACP, SACP, and control groups. Remineralization efficacy was assessed using pH-cycling, Vickers microhardness, SEM-EDX, and XRD. Antibacterial activity was evaluated using the agar diffusion method and biofilm disruption was analyzed through fluorescence microscopy against *S. mutans* and *L. acidophilus.* Statistical analysis was performed using one-way ANOVA with Tukey’s post-hoc test.

**Results:**

SEM revealed that BACP had a more uniform, densely packed structure compared to SACP. Microhardness testing showed BACP-treated samples had the highest post-treatment hardness (59.97 HV), followed by SACP (53.80 HV) and control (42.30 HV). EDX showed a higher Ca/P ratio in BACP (1.64) compared to SACP (1.54), closely resembling hydroxyapatite. BACP exhibited superior antibacterial activity in both agar diffusion and biofilm disruption analyses.

**Conclusion:**

BACP, derived from eggshell waste, demonstrated superior remineralization and antibacterial potential, making it a promising alternative to SACP for dentin repair.

**Clinical trial number:**

Not applicable.

## Introduction

Dentin demineralization plays a pivotal role in the progression of dental caries, erosion, and other pathological conditions that compromise tooth integrity. During acidic challenges caused by bacterial metabolism and dietary acids, hydroxyapatite crystals dissolve, resulting in mineral loss and the structural weakening of the dentin matrix [1]. Dentin demineralization results in increased dentin permeability, hypersensitivity, and eventual cavitation, necessitating restorative interventions if left untreated. Effective remineralization strategies are therefore essential to counteract mineral loss, restore dentin integrity, and prevent disease progression [2, 3]. 

One of the promising approaches in contemporary dentin remineralization strategies is the use of bioactive agents capable of promoting dentin remineralization. Traditional materials like fluoride, bioactive glass and various forms of crystalline calcium phosphate cements (hydroxyapatite (HAp), tricalcium phosphate (TCP), and biphasic calcium phosphate (BCP), calcium deficient hydroxyapatite (CDHA)), help to form minerals on the dentin surface or on the dentinal tubules by supporting crystal growth [4, 5]. However, they mainly cause surface remineralization i.e., on the extrafibrillar collagen surface, not within them, which is needed to mimic natural dentin structure [6]. This limitation often leads to less effective long-term results on dentin remineralization. Amorphous calcium phosphate (ACP) nano-precursors have garnered significant attention due to its high bioactivity, superior biodegradation and its ability to serve as a reservoir of calcium (Ca²⁺) and phosphate (PO₄³⁻) ions [7]. These properties make ACP as a valuable component in restorative materials such as bioactive cements, restorative materials and dentin adhesives, where it enhances the structural integrity of demineralized dentin and supports biomineralization [8]. 

The incorporation of synthetic ACP(SACP) nanoparticles into dental materials has further improved its effectiveness by enabling deeper penetration into the dentinal tubules and enhancing mechanical properties. Studies have demonstrated that ACP-containing composites and cements facilitate sustained ion release, reinforcing and improving the longevity of restorations [9, 10]. Additionally, ACP-based sealants and desensitizing agents play a crucial role in occluding dentinal tubules, reducing dentin hypersensitivity, and reinforcing dentin structure. The high surface reactivity and nanoscale dimensions of ACP particles allow for superior remineralization efficacy, positioning ACP as a key component in protecting and restoring dentin [10, 11]. 

Despite its advantages, ACP has several limitations that affect its clinical applications. One of the primary concern is its inherent thermodynamic instability, as it readily transforms into more stable crystalline phases of HAp, under physiological conditions, reducing its bioavailability and controlled ion release [12]. Thus, SACP which often transforms into HAp, cannot penetrate collagen fibrils to aid in functional remineralization and unfortunately it lack potential antibacterial properties [13]. To counteract this, stabilizing agents such as magnesium (Mg), casein phosphopeptides (CPP), and polyacrylic acid (PAA) are commonly incorporated to delay crystallization, enhance ion release, and improve adhesion to demineralized surfaces [8]. These stabilizers help sustain the amorphous state of SACP, ensuring a prolonged and controlled release of calcium and phosphate ions, which is critical for effective dentin and enamel remineralization. However, despite these modifications, maintaining long-term stability remains a challenge, necessitating further optimization to match the natural stability. Furthermore, ACP lacks intrinsic antibacterial properties, making it less effective in preventing bacterial colonization and secondary caries [14]. Moreover, the production of SACP involves complex synthesis protocols and cost-intensive processing, which may limit its widespread adoption in commercial dental products.

To address these challenges, naturally derived calcium phosphate sources have gained significant interest as viable alternatives to SACP due to their cost-effectiveness, bioactive nature along with biocompatibility. Chicken eggshells, a widely available biowaste material, are composed primarily of calcium carbonate (CaCO₃), which can be transformed into ACP through controlled thermal and chemical processing [15, 16]. The conversion of eggshell CaCO₃ into ACP provides a bioactive material capable of enhancing remineralization. Unlike SACP, which requires chemically intensive production methods, the biogenic origin of ACP (BACP) offers a more sustainable alternative while maintaining high calcium bioavailability and structural compatibility with biological tissues [17]. 

Additionally, eggshells naturally contain trace elements such as strontium (Sr), magnesium (Mg), Sodium(Na)and zinc (Zn) etc., which can play a crucial role in enhancing the bioactivity and stability of ACP [18]. Eggshell-derived BACP naturally contains these stabilizing elements, improving its stability and making it a superior choice for bone regeneration and remineralization. The presence of Mg and Sr in eggshells can significantly enhance ACP stability and its remineralization potential through a native one-pot synthesis method [19, 20]. Zinc has been reported to exhibit antibacterial properties, which may help in reducing bacterial colonization and secondary caries formation [21]. Thus, the presence of these trace elements enhances the metastability of ACP, preventing its premature crystallization and allowing for deeper penetration into demineralized dentin, leading to superior remineralization efficacy [8, 15]. 

While SACP relies on artificial stabilizers to enhance its performance, BACP offers a naturally stabilized and biologically superior alternative. This study introduces eggshell-derived BACP as a novel, sustainable alternative to synthetic ACP, to enhance native dentin remineralization and antibacterial efficacy. Its natural trace elements could enhance stability and bioactivity, offering an original, eco-friendly solution for restorative dentistry. These advantages position eggshell-derived BACP as a sustainable and functionally enhanced alternative to SACP, which can have potential applications in bioactive dental materials, desensitizing agents, and restorative formulations. This represents a significant advancement in minimally invasive restorative care for dental providers, offering a dual-function material that promotes dentin repair and inhibits cariogenic biofilm thereby enhancing both clinical outcomes and material sustainability in conservative dentistry. Initial studies suggest that eggshell-derived BACP demonstrates comparable bioactivity to SACP [16], yet a comprehensive evaluation of their remineralization potential and antibacterial activity on dentin substrates remains largely unexplored. Thus, this preliminary study aims to comparatively evaluate the dentin remineralization and antibacterial potential of SACP and BACP by assessing their ability to restore lost mineral content using microhardness test and surface elemental analysis. The proposed null hypothesis is that SACP and BACP does not have any difference in dentin remineralization and antibacterial potential.

## Methodology

### Materials used

Extracted human teeth were obtained after approval from Institutional Review Board. For BACP synthesis, chicken eggshells were collected as biowaste, cleaned, and used as a calcium precursor. Reagents such as nitric acid, diammonium hydrogen phosphate, and Tris buffer were procured from Merck (India). SACP was obtained from Sigma-Aldrich to serve as the standard comparator. Nitric acid, Diammonium hydrogen phosphate, Tris-buffer, distilled water were purchased from Merck, India. All chemicals used were of analytical grade and freshly prepared for each experiment.

### Synthesis of biogenic calcium phosphate (BACP) complex

Egg shell wastes of chicken were collected and cleaned by stripping the inner membrane which was then rinsed under water followed by overnight drying in oven at 100 °C. Samples were then powdered using agate mortar, placed in alumina crucible and heated upto 900 °C for 6 h in a furnace to obtain Calcium oxide which was then converted to calcium hydroxide solution of 1 M by dissolving it in distilled water. To this solution, nitric acid of required molar concentration was added dropwise to obtain calcium nitrate solution of 1 M. Further, diammonium hydrogen phosphate was added to get Ca/P ratio of 1.3 by maintaining the temperature below 25 °C and pH above 11. This was continued for 10 min and then the solution was centrifuged at 8000 rpm for 5 min to collect precipitate. Cold alkaline deionized water was used to wash the precipitate which was then freeze dried to obtain BACP particles [15]. 

### Characterization of SACP and BACP

The synthesized BACP powder particles were characterized to evaluate its surface morphology, elemental composition, and structural phase using Scanning Electron Microscopy with Energy-Dispersive X-ray Spectroscopy (SEM-EDX), Fourier-transform-infrared (ATR-FTIR) spectroscopy and X-ray Diffraction (XRD).For SEM-EDX analysis, a small quantity of the freeze-dried BACP powder was carefully placed on aluminum stubs using double-sided carbon adhesive tape. The samples were then sputter-coated with a thin layer of gold under vacuum using a sputter coater (Quorum Technologies) to ensure surface conductivity. High-resolution scanning electron microscopy was performed using an Apreo S SEM system (Thermo Scientific, USA) operated at an accelerating voltage of 20 kV and a working distance of 10–15 mm. Micrographs were captured at magnifications ranging from 3500X to assess particle morphology. EDX was carried out in conjunction with SEM to determine the elemental composition of the BACP powder. Spectra were acquired from three random fields on each sample, and the atomic weight percentages of calcium (Ca), phosphorus (P), oxygen (O), and trace elements such as magnesium (Mg), strontium (Sr), and carbon (C) were recorded. The Ca/P ratio was calculated based on atomic percentages to assess compositional similarity to HAp. The structural analysis of the biomaterials was performed using FTIR spectroscopy (Perkin Elmer spectrum RX1 spectrometer, Chennai, India) in the spectral range of 4000-400 cm–1 with a spectral resolution of 4 cm–1 using the KBr pellet technique. For XRD analysis, the BACP powder was finely ground using an agate mortar and placed on a flat glass sample holder. X-ray diffraction was performed using a Bruker D8 Advance diffractometer equipped with a Cu-Kα radiation source (λ = 1.542 Å), operated at 40 kV and 40 mA. The diffraction pattern was recorded over a 2θ range of 10° to 60°, with a step size of 2° and a scan speed of 6° per minute. The resulting diffractograms were analyzed for peak positions and intensities to determine the phase structure. (SRM Nano Research centre, SRMIST)

### Cell viability and proliferation

Human dental Pulp Stem Cells (hDPSCs) were commercially procured from Mothercell Biosciences Pvt Ltd, India and were cultured in DPSC BulletKit™ Medium (PT-3005, Lonza, USA) at 37 °C and 5% CO₂. Both the powders were separately diluted to 200 mg/mL in DPSC medium per ISO/EN 10993-12 guidelines, and sterilized using a 0.2-µm filter. Serial dilutions (1:1, 1:2, 1:4, 1:8) were prepared. Cytotoxicity was assessed using the MTT assay (M5655, Sigma-Aldrich, USA) at day 1, day 3 and day 7. hDPSCs (5 × 10³ cells/well) were seeded in a 96-well plate, incubated with extracts, and treated with MTT solution for 4 h. The formazan product was solubilized with acidic isopropanol, and absorbance at 570 nm was measured using a microplate reader (Tecan, Austria). Relative cell viability (%) was calculated against control (untreated cells) using optical density values. The experiments were done in triplicates and data were expressed as percentage of cell viability.

### Formulation of demineralization solution

The demineralization solution (DS) utilized for generating subsurface caries-like lesions and during pH-cycling procedures consisted of the following components: 2 mM calcium chloride dihydrate, 0.0476 mM sodium fluoride, 2.2 mM potassium dihydrogen phosphate, 50 mM acetic acid, and 10 mM potassium hydroxide. These ingredients were combined and adjusted to a pH of 5.0 [22]. 

### Preparation of artificial saliva

The artificial saliva (AS) solution was formulated by dissolving 1.25 mM calcium nitrate, 0.90 mM potassium phosphate, 129.91 mM potassium chloride, 59.93 mM tris buffer, and 2.2 g of mucin in 1 L of distilled water. The final solution was adjusted to a pH of 7.4 [23]. 

### Sample size determination

The sample size for this study was determined based on a power analysis using G*Power 3.1 software to achieve a statistical power of 80% at a 5% significance level. A minimum of 10 samples per group was required to detect a clinically relevant difference in remineralization potential between the experimental and control groups. To account for possible variations and ensure robustness, the final sample size was set at 12 specimens per group, leading to a total of 36 dentin specimens used in the study according to Madhubala et al. 2025 [17]. 

### Specimen preparation

Thirty-six extracted human mandibular molars were obtained from the patients reporting to outpatient ward of SRM Dental College, Ramapuram, Chennai according to a protocol approved by the institutional review board (SRMDC/IRB/2018/PhD/No.114) and stored in 0.2% thymol until use. Teeth with caries, cracks, cervical lesions, white spots and hypoplastic appearance were excluded from the study. All the teeth were decoronated and crowns were sectioned perpendicular to the longitudinal axis using low speed diamond saw to obtain 0.5 mm to 1 mm flat dentin slices. Markings were made on the pulpal side to identify the coronal surface and the samples were then polished using 600 grit silicon-carbide papers. Specimens were then cleaned ultrasonically in solution containing acetone, ethanol and deionized water for 10 min. the baseline microhardness and surface morphology analysis were done using Vickers microhardness test (VMH) and SEM-EDX for all the samples respectively. All dentin slabs were submerged in demineralization solution (DS) at a ratio of 20 mL per slab in Eppendorf tubes separately for a duration of 4 days. DS solution was refreshed daily and pH was closely monitored. Prior to the pH cycling regime, all samples were kept in AS. All samples were assessed using the same parameters specified previously to determine demineralization values. The samples were then randomly divided into three groups (*n* = 12 per group) Group 1– Control; Group 2– SACP; Group 3– BACP.

### pH cycling regime

All the dentin samples were subjected to pH cycling model by carrying out sequential demineralization and remineralization for 7 days according to Aruna et al.[16], [23]. Briefly, each sample underwent a pH-cycling process twice daily, involving immersion in the DS for 3 h, exposure to experimental test agents for 5 min using a microbrush with gentle strokes, and treatment with AS for 2 h at 37 °C. This was followed by overnight storage in AS. Samples were then removed from the solution and rinsed with deionized water and remineralization assessments were conducted at 7th day.

### Microhardness evaluation of dentin

Microhardness values were determined for all dentin sample surface using a Vickers microhardness tester (DUH-211; Zwick, Germany) under a 20 g load for 10 s (Liang et al., 2019). Three indentations were made on each dentin surface: starting at the center, then moving left and right, maintaining a 100 μm gap between each indent. A subsequent set of indentations was placed so as not to overlap the initial indent. The three readings were taken and averaged to produce a single microhardness value per sample. Baseline (BMH), demineralized (DMH), and remineralized (RMH) microhardness values were taken at corresponding time as mentioned above and tabulated for statistical analysis.

###  Dentin tubule occlusion (DTO) and surface element analysis using SEM-EDX 

SEM-EDX was employed to evaluate dentin tubule occlusion (DTO) and perform surface element analysis. For this study, three dentin discs from each group were selected and prepared for imaging. The samples were first dried in a vacuum desiccator for 24 h before being mounted on aluminium stubs. To enhance conductivity, the discs were sputter-coated with gold and subsequently examined under SEM at a magnification of 3500× for qualitative morphological analysis on dentin tubular occlusion. The percentage of occluded tubules was quantified using image analysis software (Adobe Photoshop, Version 12.4, SJ, USA). Measurements were performed on micrographs captured 3500x magnification, where the area occupied by partially or completely occluded tubules was calculated relative to the total tubule area [21]. For each specimen, images from five distinct regions were analyzed, and the mean value across 12 specimens was computed to obtain the final percentage of DTO.

In addition to structural evaluation, elemental microanalysis was conducted using EDX to characterize the chemical composition of the dentin surface. This analysis was performed on three distinct sample areas, maintaining a working distance of 15 mm, an acceleration voltage of 25 kV, and a magnification of 500x. The degree of remineralization was assessed by determining the calcium and phosphorus content, from which the calcium/phosphate ratio was derived. This ratio serves as an indicator of the extent of remineralization within the dentin matrix.

### Crystal deposit analysis

To evaluate HAp formation, XRD was performed on dentin samples after treatment. Parameters included Cu-Kα radiation (λ = 1.542 Å), a tube current of 40 mA, tube voltage of 40 kV, a scan speed of 6°/min, and a scan range of 10°–60°. Characteristic peaks at 35° and 38° (211 and 310 planes) were used to assess crystalline conversion. Each sample was scanned in duplicate to ensure reproducibility.

### Antibacterial evaluation

#### Agar diffusion test

To assess the antimicrobial efficacy, S. mutans was inoculated onto agar plates (*n* = 6) using sterile swabs moistened with bacterial suspensions containing 10⁸ CFU/mL. The swabs were brushed across the agar surface in a zigzag motion, starting from the top-left corner and ending at the bottom-right corner to ensure uniform bacterial spread. Wells measuring approximately 6 mm in diameter were aseptically created at the center of each agar plate using a sterile punch. A fixed volume of different dilutions (1;1, 1:2, 1:4 and 1:8) of the experimental samples was carefully pipetted into the wells. The plates were subsequently incubated at 37 °C for 48 h under optimal conditions. Following incubation, zones of inhibition (ZOI) appeared as clear, circular, or oval regions around the wells, indicating bacterial growth suppression. The shortest diameter (D1) and the longest diameter (D2) of each inhibition zone were measured, and the average of these values was recorded as the ZOI diameter. All experiments were conducted in triplicate for consistency and accuracy.

#### Analysis of biofilm disruption through fluorescent staining

Thirty dentin samples were prepared by cleaning, autoclaving, and storing them in sterile PBS until further use. To mimic oral conditions, the samples were placed in 24-well plates and coated with either saliva or an artificial pellicle. *Streptococcus. Mutans (S mutans)* and *Lactobacillus acidophillus (L.acidophillus)* cultures, grown in BHI broth with 1% sucrose and adjusted to 0.5 McFarland standard (1.5 × 10⁸ CFU/mL), were applied to the dentin samples and incubated for one week at 37 °C in a 5% CO₂ atmosphere to promote biofilm formation. After biofilms formed, the samples were exposed to dilutions of various experimental agents (1;1, 1:2, 1:4 and 1:8) for 5 min for 7 days and incubated at 37 °C. Following treatment, the samples were rinsed thoroughly to remove unattached bacteria. Sessile bacteria on the samples were stained by immersing them in a solution containing acridine orange and ethidium bromide (AO/EtBr) for 30 min at 37 °C in the absence of light. The stained samples were examined using a fluorescent microscope at 40x magnification. Live bacteria with intact membranes fluoresced green, while dead bacteria with compromised membranes displayed red to yellow fluorescence. The fluorescence intensities of both red (non-viable bacteria) and green (viable bacteria) were quantified using Image Examiner J software. These values were then used to calculate the percentage of bacterial reduction.

#### Statistical analysis

All statistical analyses were performed using IBM SPSS Statistics for Windows, Version 29.0 (IBM Corp., Armonk, NY, USA). Prior to conducting parametric tests, the normality of data distribution for all continuous variables including MTT assay, MHV, DTO, and antibacterial measurement was assessed using the Kolmogorov–Smirnov test. Homogeneity of variances was evaluated using Levene’s test to ensure assumptions for ANOVA were met. For intergroup comparisons of MHV and antibacterial activity assessments among the three groups one-way Analysis of Variance (ANOVA) was conducted. When significant differences were found (*p* < 0.05), Tukey’s HSD post-hoc test was applied to identify specific group differences. Data from each dilution level in antibacterial assessment was analyzed separately to identify concentration-dependent effects. In the case of MTT assay results, data from five conditions (four experimental dilutions and one control) were analyzed using two-way ANOVA to examine the interaction between treatment type (SACP vs. BACP) and time (Day 1, Day 3, and Day 7). Post-hoc comparisons were conducted using Bonferroni correction to control for multiple testing. Additionally, independent sample t-tests were used to compare cytotoxicity differences between the two groups at each time point and dilution, when normality was assured. All quantitative data were reported as mean ± standard deviation (SD). A p-value < 0.05 was considered statistically significant in all tests.

## Results

### Characterization of SACP and BACP

**Fig. 1:**
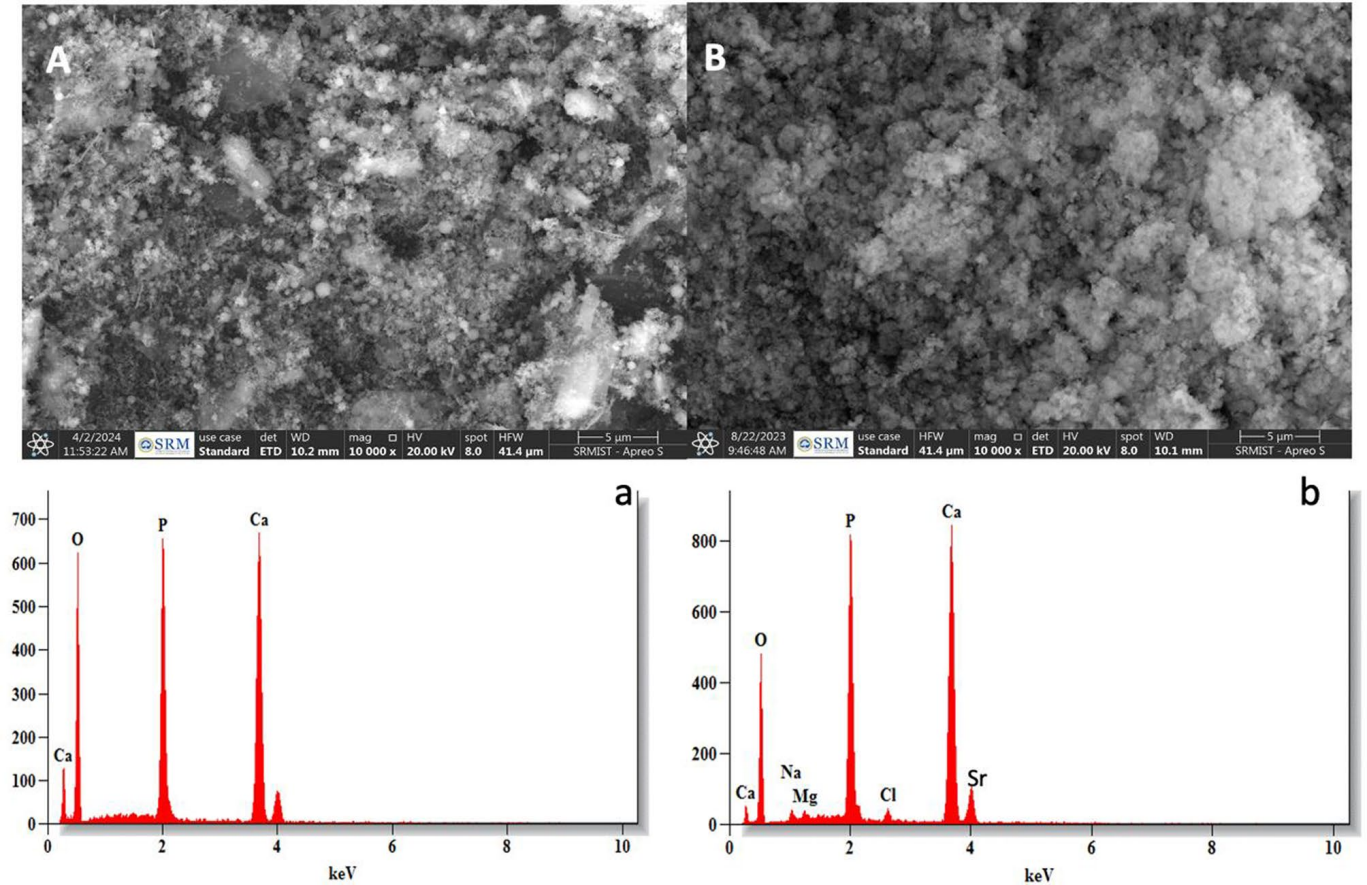
Representative SEM- EDX of **A**, **a**) Synthetic amorphous calcium Phosphate (SACP); **B**, **b**) Biogenic amorphous calcium phosphate (BACP)

The SEM analysis reveals that SACP (Fig. 1A) consists of irregular, loosely packed agglomerates with a heterogeneous morphology, indicating a less compact structure. In contrast, BACP (Fig. 1B) displays a more uniform and densely packed arrangement of smaller spherical particles, suggesting a more organized and controlled formation. EDX analysis confirmed the presence of Ca, P, and O in both materials. BACP additionally contained trace elements such as C, Mg, and Sr, along with carbonate (CO₃²⁻), distinguishing it from SACP. The elemental distribution in BACP appeared more varied, indicating a non-uniform composition, whereas SACP exhibited a relatively homogenous elemental dispersion.( (Fig. 1a, b).

**Fig. 2:**
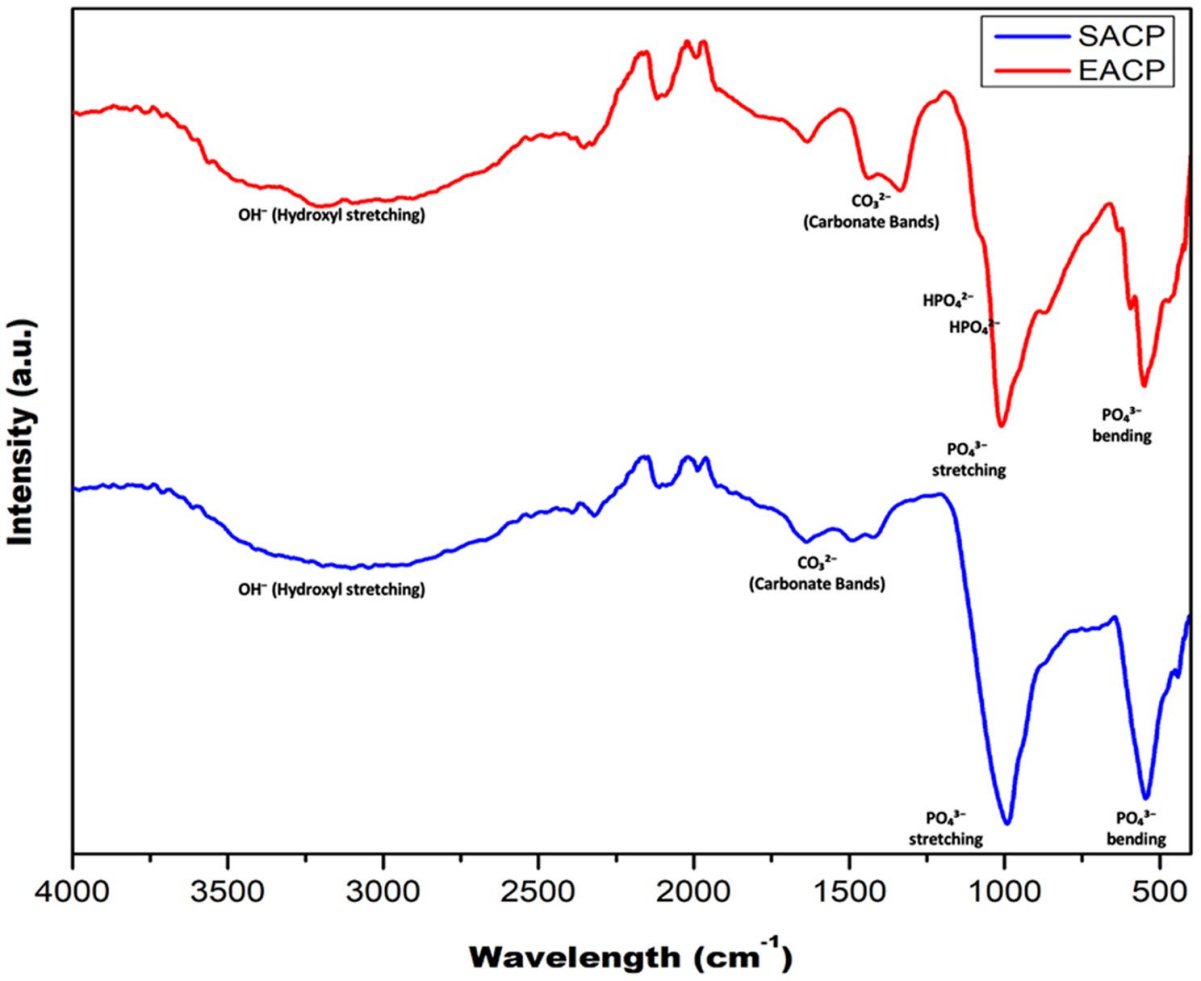
FTIR image of A) Synthetic amorphous calcium Phosphate (SACP); B) Biogenic amorphous calcium phosphate (BACP)

FTIR spectra of SACP and BACP revealed key similarities and differences. Both materials exhibit characteristic phosphate (PO₄³⁻) vibrations, with strong bands in the 1040–1100 cm⁻¹ region (asymmetric stretching) and 550–600 cm⁻¹ (bending modes), confirming their phosphate composition. Broad hydroxyl (OH⁻) bands around 3200–3600 cm⁻¹ indicate water incorporation, which is more pronounced in BACP, likely due to its biological origin and greater hydration. The presence of carbonate (CO₃²⁻) bands near 1400–1450 cm⁻¹ is more distinct in BACP, suggesting a degree of carbonate substitution within the phosphate lattice, a feature typical of BACP. Both spectra exhibit broad peaks, confirming their amorphous nature, though the intensity differences indicate slight variations in chemical composition and hydration. These findings suggest that while both SACP and BACP share fundamental calcium phosphate characteristics, BACP retains biogenic components such as higher carbonate substitution and water content, which could influence its reactivity and bioavailability compared to purely synthetic forms (Fig. 2).

**Fig. 3:**
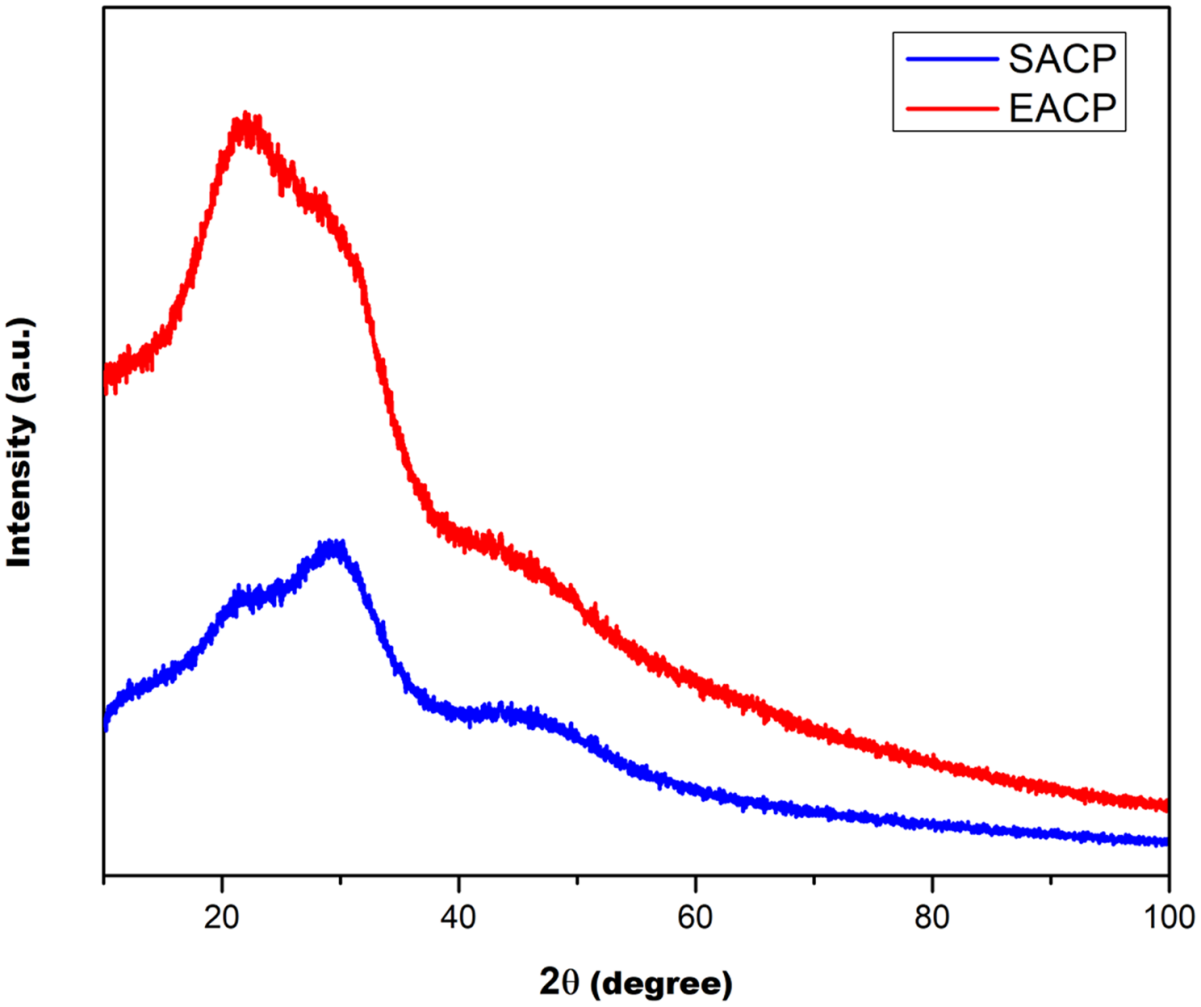
XRD spectra of A) Synthetic amorphous calcium Phosphate (SACP); B) Biogenic amorphous calcium phosphate (BACP)

The XRD patterns of BACP and SACP showed broad amorphous peaks, confirming their non-crystalline nature. The absence of sharp peaks indicates a lack of well-ordered crystalline structures, suggesting that both materials exist in an amorphous phase (Fig. 3).

### Cell viability evaluation

**Fig. 4:**
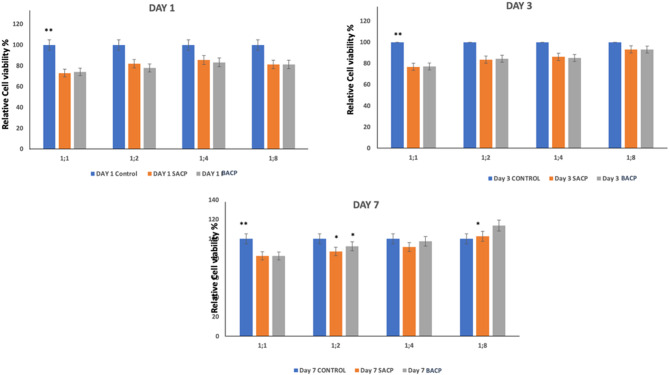
Relative cell viability of hDPSCs exposed to Control, SACP, BACP. Results are expressed as relative cell viabilities (percentage of control). **A**) 24 h and **B**) 48 h **C**) 72 h. Data shownare the mean (± SD) of four replicates. significant differences compared to the control are marked as * *p* < 0.05, ** *p* < 0.01

Cell viability trends (Fig. 4) reveal significant differences between the two materials, particularly at higher dilutions and extended time points. On intergroup comparison of relative cell viability percentage showed that on Day 1, there was no significant difference between SACP and BACP at 1:1, 1:2, and 1: dilutions. However, at 1:8, BACP had significantly higher cell viability. On Day 3, no significant differences were observed at any dilution (*p* > 0.05). On Day 7, BACP showed significantly higher cell viability at 1:2 and 1:8 while 1:4 approached significance (*p* = 0.060). The control group remained unchanged across all days. On intragroup comparison, both SACP and BACP showed an increasing trend in relative cell viability percentage over time. In SACP, cell proliferation gradually increased across days, especially at higher dilutions, with significant differences (*p* < 0.05). BACP also showed a consistent rise, with the highest increase observed at 1:8 dilution on Day 7 (*p* = 0.000), indicating a stronger biocompatibility effect compared to SACP.

### Microhardness evaluation

The Kolmogorov-Smirnov (K-S) test showed that all groups had a normal data distribution (Sig > 0.05). A one-way ANOVA test found significant differences (*p* = 0.000) between all the groups. All the groups revealed a significant decrease in MHV following demineralization. The control group showed the least improvement after remineralization, with only a modest change. SACP and BACP groups demonstrated substantial remineralization, with BACP exhibiting the highest MHV with statistically significant difference. Multiple comparison tests confirmed significant post-treatment differences (*p* = 0.000), with BACP showing the highest remineralization effect. These findings indicate that both experimental groups were effective in enhancing remineralization, with BACP showing the greatest potential for restoring dentin microhardness (Table 1).

**Table 1:** Showing mean values of baseline microhardness, microhardness values after demineralization and remineralization of various groups( * *p* < 0.05)

GROUP	FOLLOW UP	MEAN MHV	S.D	95% CONFIDENCE INTERVAL		ANOVA	SIG
				LOWER	UPPER		
BASELINE	CONTROL	63.90	4.564	60.63	67.16	56.473	0.087
	SACP	59.23	4.700	55.86	62.59		
	BACP	60.80	4.521	57.56	64.03		
DEMIN	CONTROL	34.95	3.351	32.55	37.35	145.706	0.006*
	SACP	28.34	6.380	23.77	32.90		
	BACP	34.95	4.250	31.90	37.99		
POST TREATMENT	CONTROL	42.30	3.248	39.97	44.62	804.246	0.000*
	SACP	53.80	5.770	49.67	57.92		
	BACP	59.97	2.508	58.17	61.767		

### SEM EDX Anlaysis

**Fig. 5:**
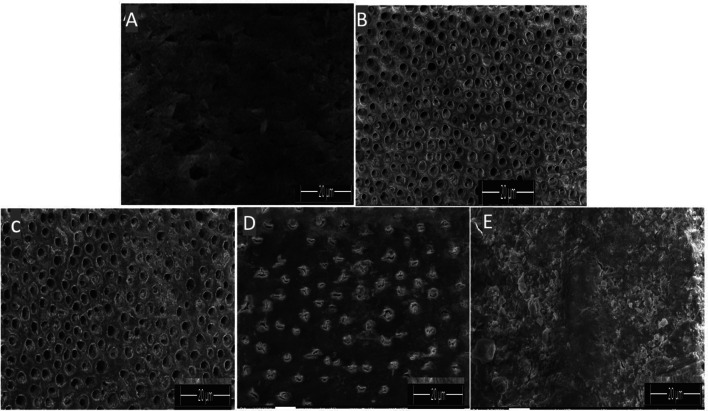
Representative SEM image of dentin occlusion by various experimental agents **A**) Sound dentin; **B**) Demineralized Dentin **C**) control: **D**) SACP; **E**) BACP(3500x)magnification

SEM-EDX evaluation revealed distinct differences between the groups. The sound tooth and (Fig. 5A) exhibited a smooth, well-preserved surface with minimal irregularities, indicating an intact enamel or dentin structure. In contrast, demineralized dentin (Fig. 5B) under SEM appears as a porous, rough surface with widened dentinal tubules and exposed collagen fibrils due to loss of mineral content. After treatment, The control group (Fig. 5C) displayed noticeable surface demineralization, characterized by roughness and porosity, suggesting significant mineral loss. After immersion in SACP (Fig. 5D), the surface showed partial remineralization, with smaller, more uniform crystals, indicating some recovery, though it did not fully restore the surface to the level of the sound tooth. However, after immersion in BACP (Fig. 4E), the specimens demonstrated the most substantial remineralization, with larger and denser mineral deposits, closely resembling the structure of the sound tooth. These SEM-EDX results indicate that BACP was the most effective in restoring mineral content and surface integrity compared to both SACP and the control group.

**Fig. 6:**
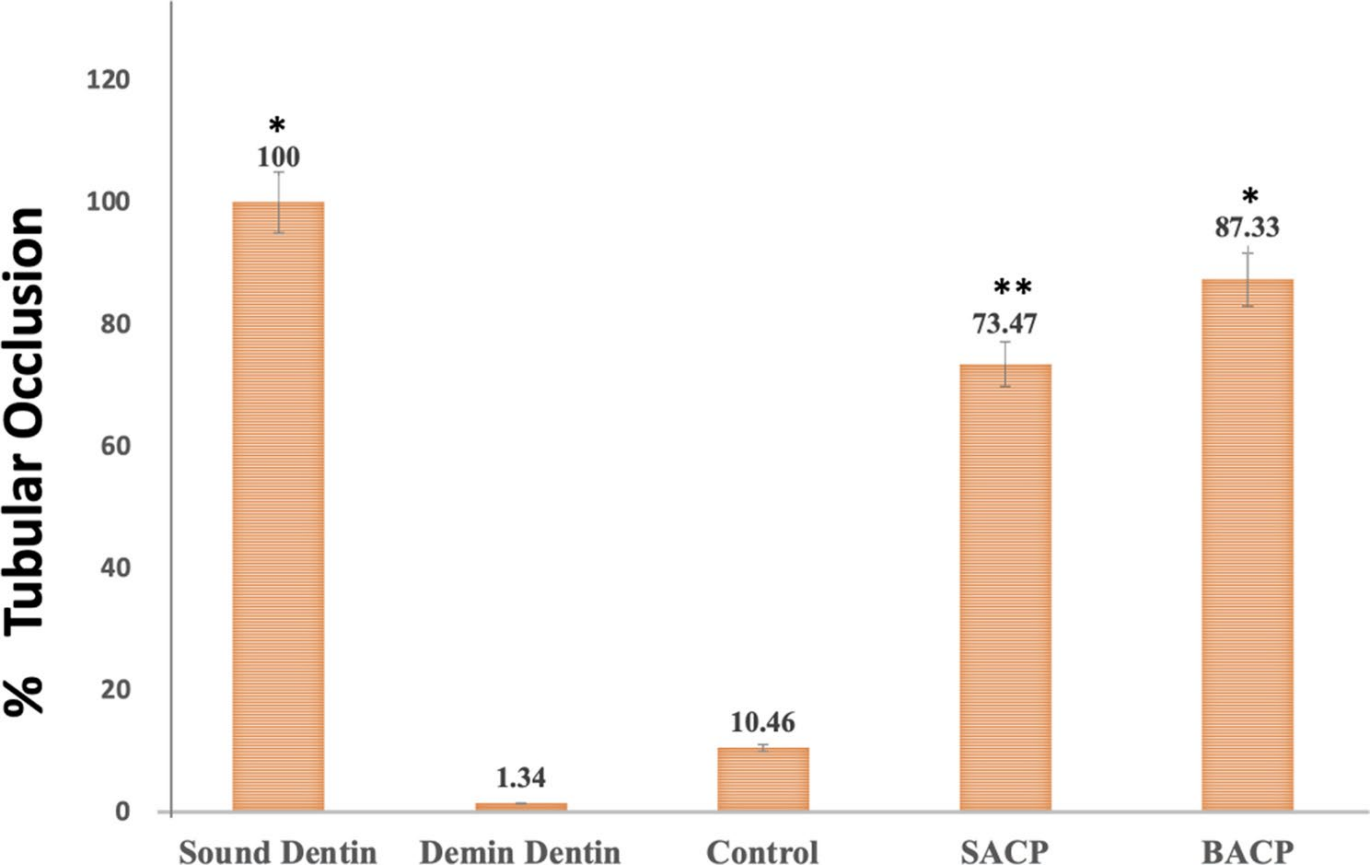
Intergroup comparison of dentinal tubular occlusion (%). The bar graph depicts the percentage of tubular occlusion observed in Control, SACP, and BACP groups.(* denotes the statistical significant difference exists)

Pairwise intergroup comparisons (Table 2; Fig. 6) revealed statistically significant differences in the percentage of tubular occlusion among the tested groups. Both SACP and BACP groups showed significantly higher tubular occlusion compared to the Control group (*Control vs. SACP*: Test statistic = -40.0, *p* < 0.001; *Control vs. BACP*: Test statistic = -60.0, *p* < 0.001). Although the BACP group exhibited a higher mean occlusion percentage than SACP, their difference was marginally significant (*SACP vs. BACP*: Test statistic = -20.0, *p* = 0.049).

**Table 2:** Intergroup pairwise comparison on % Tubular occlusion

Groups	Test Statistic	Std. Error	Std. Test Statistic	Sig.
Control-SACP	-40.0	10.997	-3.637	< 0.001
Control-BACP	-60.0	10.997	-5.456	< 0.001
SACP-BACP	-20.0	10.997	-1.819	0.049

**Fig. 7:**
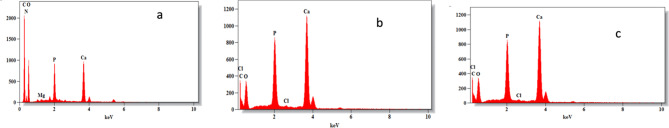
EDX image of dentinal deposits by various experimental agents **A**) Sound dentin; **B**) control: **C**) SACP; **D**) BACP

In the EDX spectra (Fig. 7), demineralized sample shows lower calcium (Ca) and phosphorus (P) peaks, indicating the loss of mineral content. In contrast, the ACP-treated sample exhibits significantly higher Ca and P peaks, confirming successful remineralization. A higher Ca/P ratio was attained in dentin surfaces immersed in SACP and BACP groups compared to the control group among the experimental agents BACP exhibited a Ca/P ratio of 1.64, while SACP showed a comparatively lower ratio of 1.54. The higher Ca/P ratio in BACP suggests a composition that is more similar to HAp (Table 3).

**Table 3:** EDX intensity spectra of inorganic components

Dentin treatment	Atomic weight%		Ca/P ratio Mean ± SD	P value
	P	Ca		
Intact	7.35	12.10	1.64 + 0.03	< 0.05*
Demineralized	2.73	4.43	1.41 + 0.33	
Control	14.89	32.77	2.10 + 0.11	
SACP	6.90	9.67	1.54 + 0.06	
BACP	5.40	8.74	1.64 + 0.67	

### XRD analysis

**Fig. 8:**
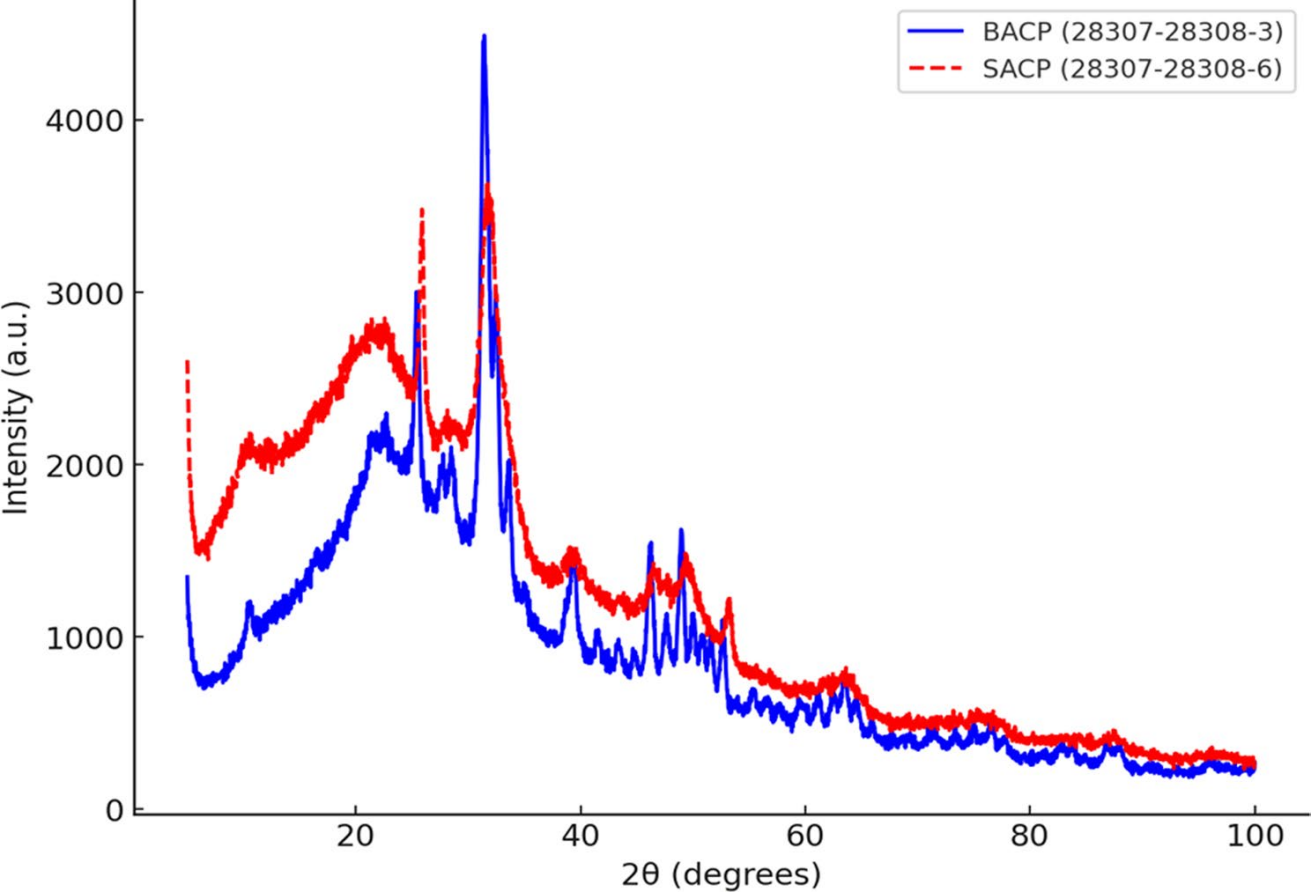
XRD plot of Biogenic ACP (BACP) corresponds to the blue curve, and Synthetic ACP (SACP) corresponds to the red curve

XRD analysis (Fig. 8) confirms the presence of characteristic HAp peaks at 35° (211 plane) and 38° (310 plane) in both BACP and SACP samples, as referenced in standard JCPDS data. The BACP sample exhibits peak intensities of 1619 at 35° and 1344 at 38°, while the SACP sample shows slightly lower intensities of 1551 at 35° and 1196 at 38°, indicating differences in crystallinity. The higher peak intensities in the BACP sample suggest a greater degree of HAp crystallization potential than SACP.

### Antibacterial evaluation

#### Zone of Inhibition

**Fig. 9:**
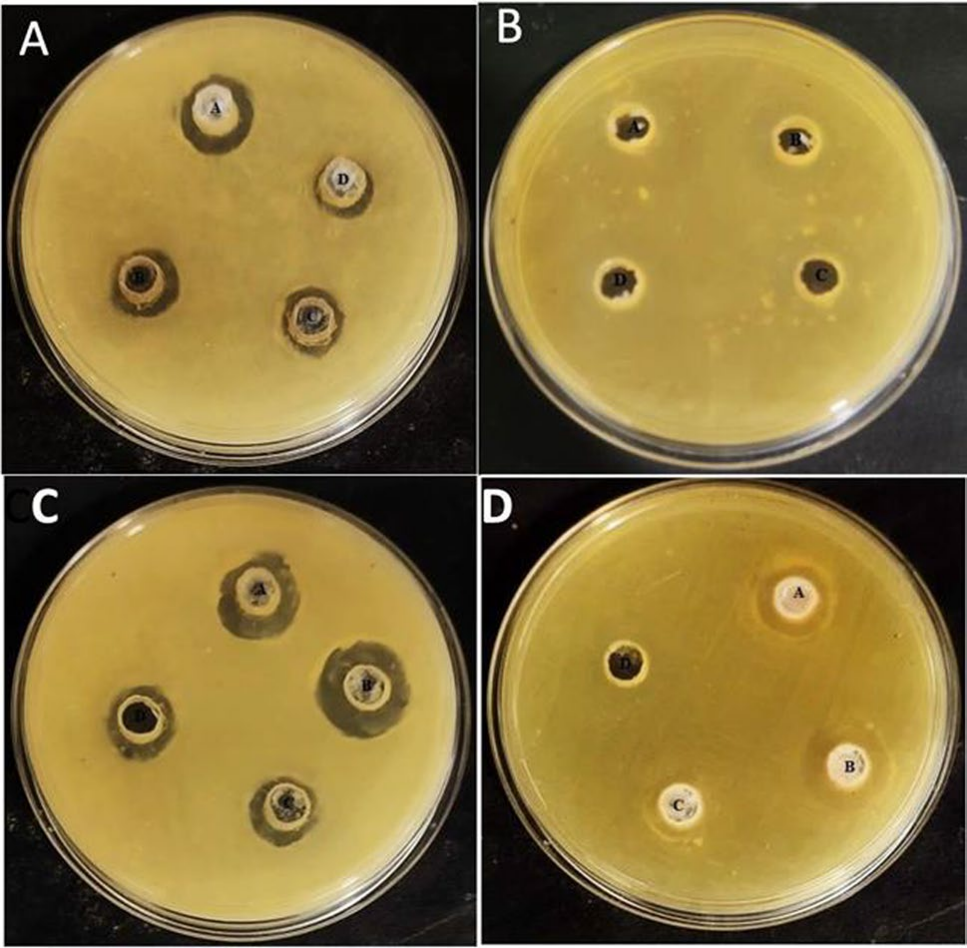
Zone of inhibition on Agar diffusion test by various groups **A**) SACP against *S.mutans*; **B**) SACP against *L. acidophillus* BACP; **C**) BACP against *S.mutans***D**) BACP against *L. acidophillus* BACP at 1:1,1:2, 1:4, and 1:8 dilutions

**Fig. 10:**
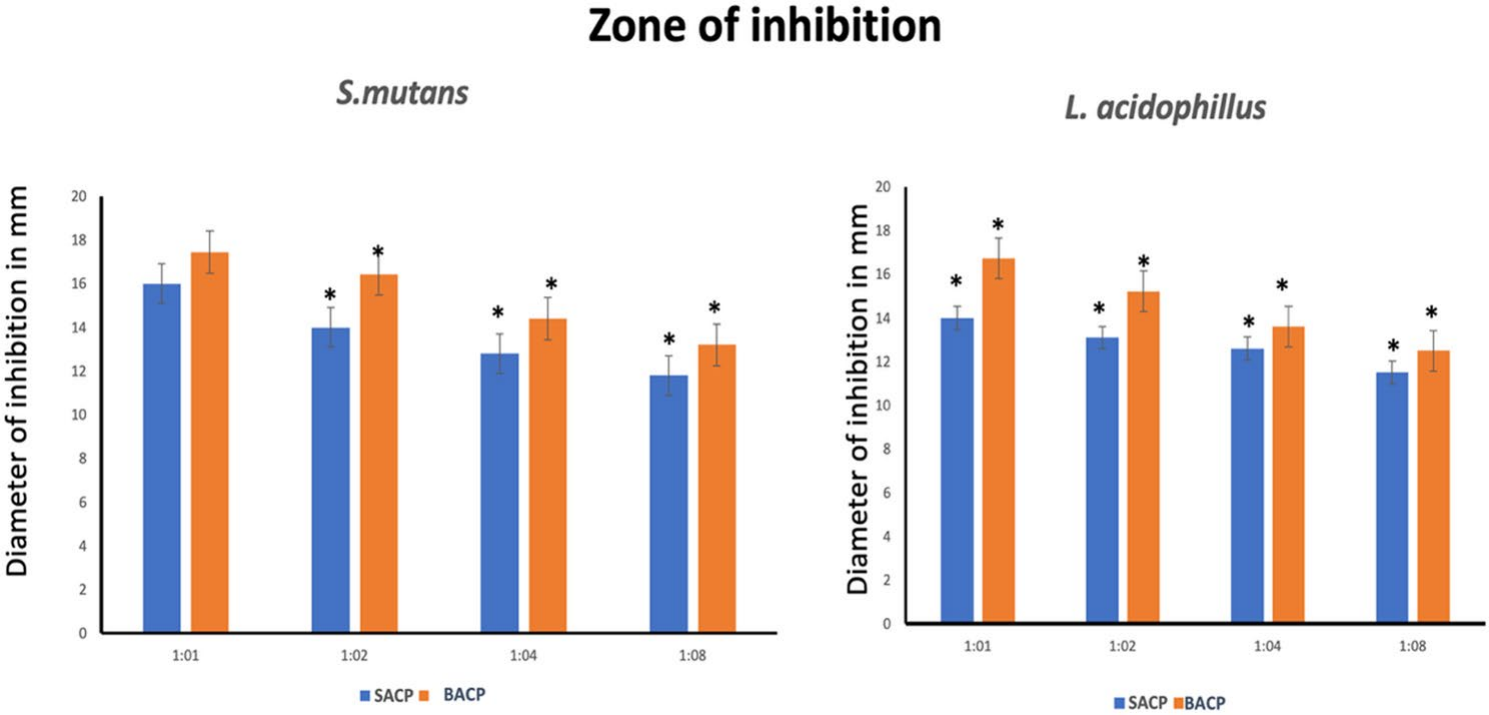
Graphical representation of Zone of inhibition (mm) on agar diffusion test by various groups **A**) against *S.mutans*; **B**) against *L. acidophillus* BACP at 1:1,1:2,1:4, and 1:8 dilutions (*denotes statistical difference)

The quantitative data for the zone of inhibition is presented in Figs. 9 and 10, which compares the antibacterial activity of two formulations: SACP (blue bars) and BACP (orange bars) against *S. mutans* and *L. acidophilus* at different dilutions (1:1, 1:2, 1:4, and 1:8). For *S. mutans*, BACP consistently demonstrated a higher zone of inhibition across all concentrations compared to SACP. The maximum inhibition zone was recorded for the 1:2 concentration, where BACP showed a statistically significant difference compared to SACP (*p* < 0.05). Similarly, for *L. acidophilus*, BACP exhibited superior antimicrobial activity at all concentrations, with the highest inhibition recorded at 1:1 and 1:2 concentrations, indicating significantly greater efficacy than SACP.

#### Biofilm disruption potential

**Fig. 11:**
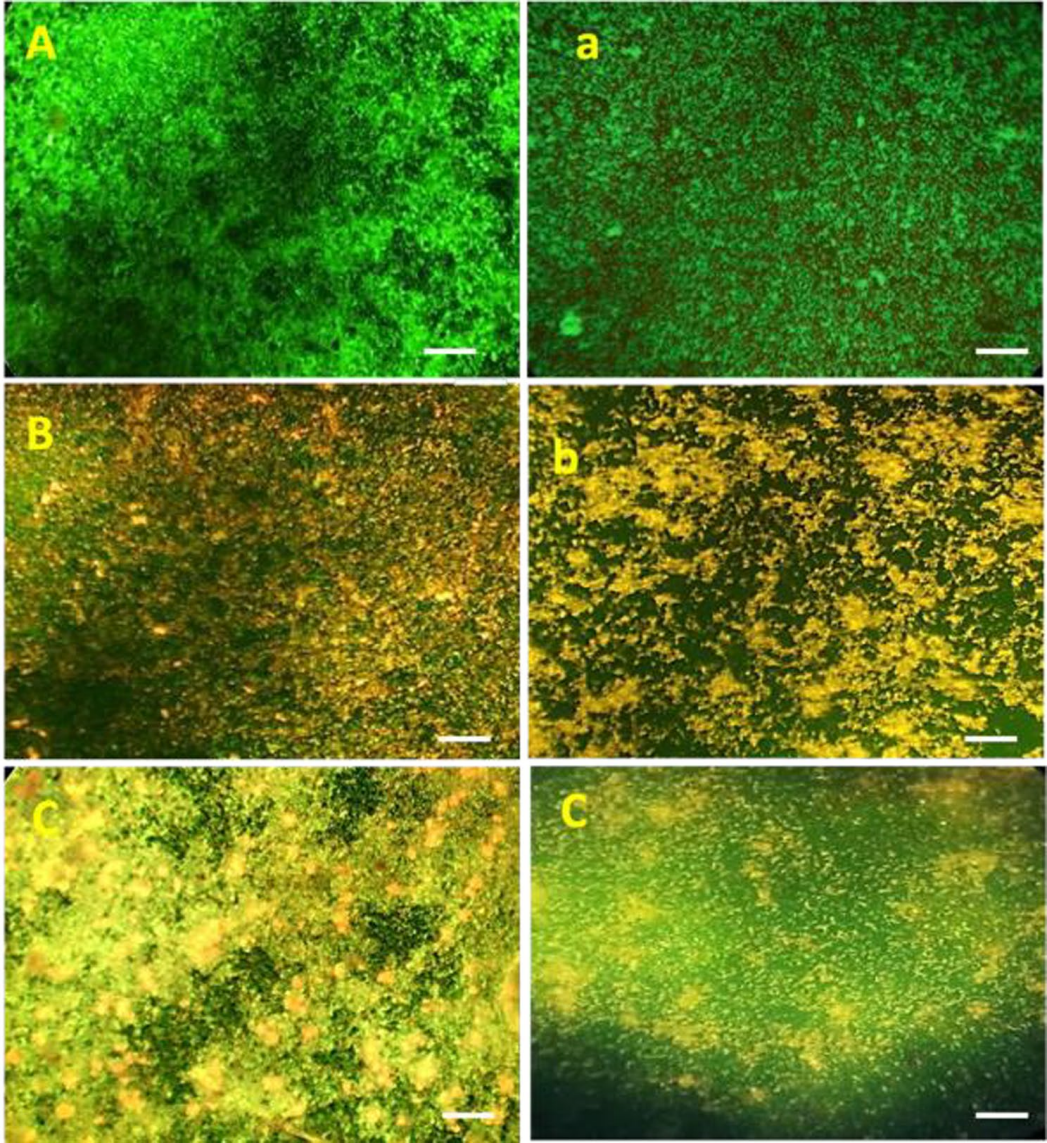
Representative pictures of fluorescent staining of viable and apoptotic bacteria exhibited by various groups **A**) Control group against *S.mutans*; **a**) Control group against *L. acidophillus*** B**) SACP against *S.mutans*** b**) SACP against *L. acidophillus*; **C**) BACP against *S.mutans****c****)* BACP against *L. acidophillus* BACP at 1:1 Scale bar represents 100 μm

**Fig. 12:**
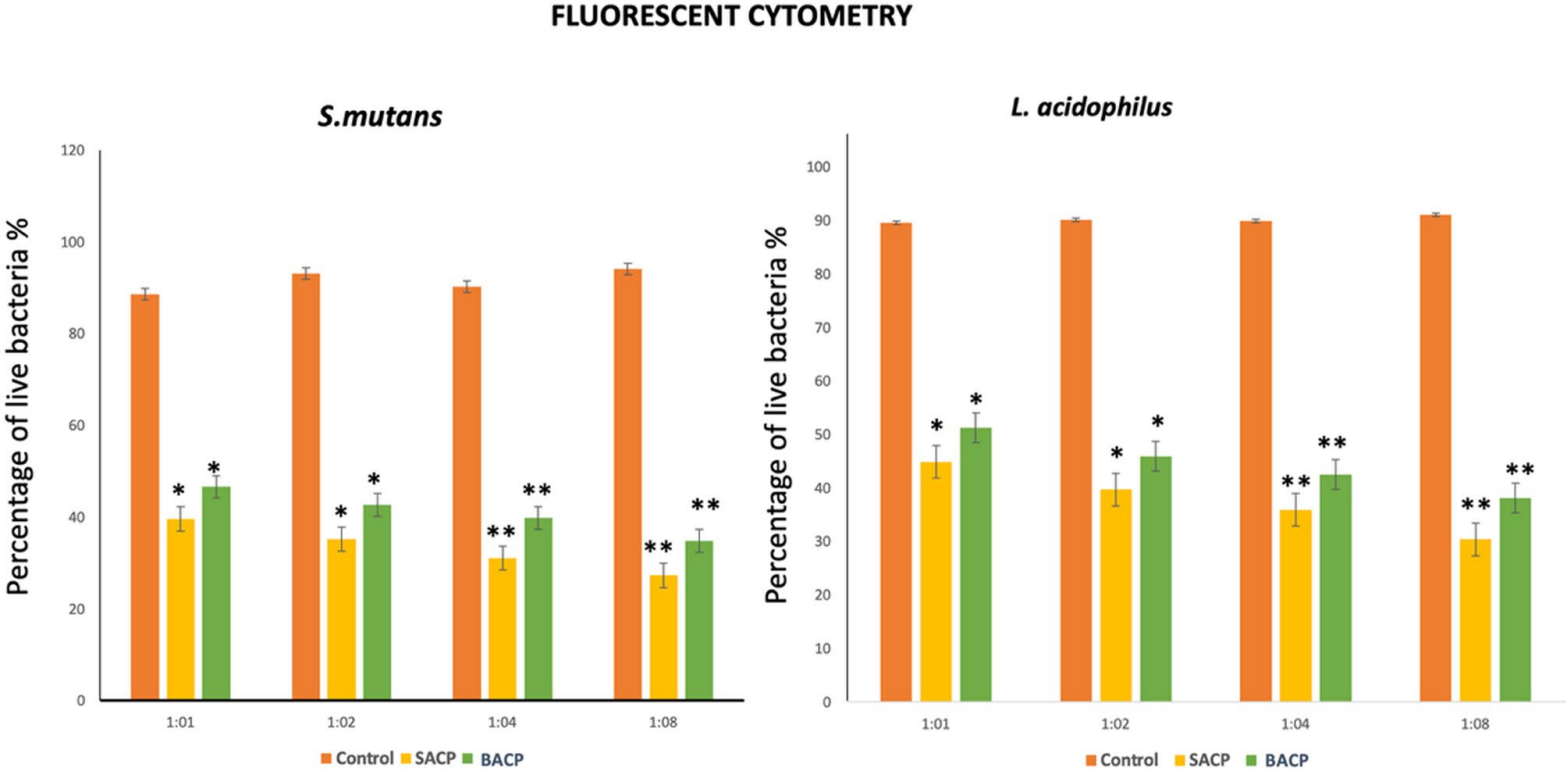
Graphical representation of percentage of live bacteria in fluorescent cytometry assay by various groups **A**) against *S.mutans*; **B**) Against *L. acidophillus* BACP at 1:1,1:2,4, and 1:8 dilutions (statistical significant difference of *p* < 0.05 and *p* < 0.01 were denoted as (*) and (**))

The comparative analysis using fluorescence microscopy (Fig. 11 and 12) clearly demonstrated that BACP exhibited superior antibacterial efficacy compared to SACP. The results showed a significantly higher reduction in bacterial viability and biofilm disruption against both *S. mutans* and *L. acidophilus* after BACP treatment at all dilutions. (*p* < 0.05(1:1,1:2) and *p* < 0.01(1:4,1:8)) It was confirmed a higher percentage of dead bacterial cells with BACP, suggesting its enhanced ability to disrupt biofilms and inhibit bacterial growth more effectively.

## Discussion

The study employed a systematic approach to compare the structural and elemental differences between synthetic and biogenic forms of ACPs. The characterization provided comprehensive insights into the physicochemical properties of both SACP and BACP. The synthesis of BACP from eggshell-derived calcium carbonate was carried out using a controlled simple precipitation method, which ensured the formation of amorphous calcium phosphate. SACP, on the other hand, was commercially obtained and used as a comparative standard. The characterization of BACP and SACP reveals distinct structural and compositional differences that influence their functional properties. Morphological analysis indicates that BACP exhibits a smaller, more irregular particle size compared to SACP, which may enhance surface reactivity and ion release for remineralization applications [12, 16]. The higher porosity of SACP suggests a faster dissolution rate, potentially affecting its bioavailability.

FTIR analysis confirms the presence of characteristic phosphate (PO₄³⁻) and carbonate (CO₃²⁻) functional groups in both the materials, with BACP displaying more pronounced carbonate bands. This suggests a biomimetic composition closer to natural HAp, which may enhance biocompatibility and mineralization potential [24]. The broader hydroxyl (OH⁻) stretching bands in BACP indicate higher structural modification, reinforcing its amorphous nature [24, 25]. XRD analysis further supports the amorphous structure of both materials, with BACP exhibiting lower crystallinity. The elemental composition confirms the presence of trace ions in BACP, which might have contributed to improved bioactivity and controlled remineralization kinetics. These elemental differences suggest that BACP may exhibit enhanced biocompatibility and functionality in the biological environment of cell seeding. The implications of these findings are significant in the context of biomaterial applications [26]. A higher Ca/P ratio, as observed in BACP could enhance the osteoconductive and osteoinductive properties, making it a promising candidate for bone regeneration and repair also [27]. Additionally, the presence of biologically relevant components in BACP can also improve its resorption behaviour and integration with natural bone tissue However, SACP also can still serve as a valuable material for biomedical applications, particularly in cases where controlled dissolution or specific ion release is desired [28, 29]. 

ACP is widely studied for its ability to act as a transient precursor to HAp, facilitating the remineralization of demineralized dentin by releasing bioavailable calcium and phosphate ions. Cell viability of SACP and BACP is a critical factor in their potential use for dentin remineralization. This structural feature enhances its ability to transform into bioactive HAp upon interaction with physiological fluids. The lower viability of SACP compared to BACP may be due to faster dissolution, leading to a burst release of calcium and phosphate ions, causing pH fluctuations and osmotic stress [30]. Unlike BACP, which contains organic components that regulate ion release and enhance cell differentiation and interaction, SACP lacks these bioactive molecules, reducing its stability [31]. Additionally, poor nucleation control and higher surface reactivity in SACP could lead to ROS generation which further attributes to the impact on cell viability [32]. These factors highlight superior cytocompatibility of BACP compared to SACP.

The results of microhardness evaluation revealed a significant change in VMH values following across all groups. Following demineralization, the microhardness values decreased considerably in all groups, confirming mineral loss. BACP demonstrated superior VMH values compared to SACP, indicating enhanced mechanical stability [24]. The higher microhardness gained by BACP group attributes to the more compact structural arrangement and more controlled mineralization process. The differences observed between BACP and SACP can also be attributed to the inherent variations in their synthesis and formation environments. BACP, being derived from biological sources, is likely to contain organic matrix components that influence its mineral composition and enhance its bioactivity. The trace elements like magnesium (Mg) and strontium (Sr), known to enhance remineralization and stability of amorphous calcium phosphate phases [33–36]. Additionally, magnesium inhibits the rapid transformation of ACP into HAp, allowing for prolonged ion release, which may contribute to the sustained increase in microhardness observed in the BACP group [36]. These organic components may contribute to the nucleation and growth of calcium phosphate, leading to a more balanced Ca/P ratio and improved biomimetic properties [37, 38]. Conversely, SACP might lack these biological influences, resulting in a structure with a slightly lower Ca/P ratio and potentially different physicochemical characteristics.

This was even more evidenced by SEM imaging which revealed distinct structural differences between the. groups. The control group displayed noticeable surface demineralization, characterized by increased porosity and exposure of dentinal tubules. The increased homogeneity in BACP-treated surfaces could be attributed to the improved remineralization process, likely due to the presence of carbonate (CO3²⁻) and other trace elements that enhance mineral deposition and tubule occlusion [39]. Quantitatively, BACP achieved the highest percentage of tubular occlusion (87.33%), significantly surpassing the control and SACP groups, suggesting superior efficacy in restoring dentin integrity and reducing hypersensitivity potential.

Similarly, BACP group exhibited the highest Ca/P ratio (1.64) in EDX analysis, aligning closely with the stoichiometry of HAp, which typically ranges from 1.5 to 1.67 [40]. suggesting a more stable and biologically relevant mineral phase. This suggests that BACP might possess a higher degree of crystallization from a more ordered amorphous structure that facilitates a closer resemblance to natural bone mineral. In comparison, the SACP-treated group showed a lower Ca/P ratio (1.54), indicating a less mature mineral deposition and it implies a composition with a greater presence of phosphate relative to calcium, which might affect its stability and biological interactions. Moreover, the presence of trace elements such as Sr and Mg in BACP further supports its enhanced remineralization efficacy, as Sr has been shown to replace Ca in HAp, improving mechanical strength and bioactivity [24, 41–44]. These findings suggest that BACP provides a more mineral deposition which is more of structural similarity with HAp.

This was confirmed by XRD analysis that BACP exhibited higher crystallinity compared to SACP, suggesting a more effective transformation of amorphous calcium phosphate into HAp. This enhanced crystallization may be attributed to the bioactive nature of BACP, which provides favourable conditions for nucleation and phase transformation. The structural and compositional differences between BACP and SACP likely influence ion diffusion and stability, affecting the crystallization process [15, 16]. These findings align with previous studies highlighting the role of bio-originated calcium phosphates in promoting mineralization [24, 41, 45, 46]. The results suggest that BACP may be a more suitable candidate for applications in dentin remineralization and other dentin regeneration therapies due to its superior HAp-forming ability formulations.

The antibacterial property of SACP is often limited, making it less effective against bacterial biofilms, which plays the major factor in dental caries progression [7, 28]. In contrast, BACP, derived from eggshell waste, was shown to have superior antibacterial action and biofilm disruption potential. The biogenic nature of BACP allows for a more controlled release of ions and improved interaction with bacterial cell membranes, making it more effective in preventing bacterial growth and biofilm formation [47]. The superior performance of BACP could be attributed to its surface characteristics and higher bioactivity, which enhances bacterial cell wall penetration and disrupts biofilm integrity. The uniform and densely packed crystal structure of BACP, as opposed to the irregular particles in SACP, contributes to its enhanced antibacterial effects [14]. Moreover, the ability of BACP to inhibit bacterial adhesion and biofilm formation has been demonstrated in previous studies, making it a promising alternative for dental applications where antimicrobial properties are critical. In addition to its structural advantages, BACP contains trace elements such as Sr and Zn, which enhance its antibacterial activity [48]. Strontium ions have been shown to inhibit bacterial adhesion and biofilm formation by altering bacterial membrane integrity and disrupting metabolic processes [43]. Similarly, zinc ions exert strong antibacterial effects by interfering with bacterial enzyme systems, inhibiting glycolysis, and destabilizing bacterial cell membranes [48]. The synergistic action of these trace elements with calcium and phosphate further strengthens the antimicrobial efficacy of BACP, making it a promising alternative to SACP for preventing bacterial growth and enhancing dentin remineralization. Thus, the presence of these trace elements in BACP improves its antibacterial action and enhances its remineralization potential, making it a dual-functional material for dental applications.

The combined results of microhardness, SEM-EDX, XRD and antibacterial activity indicate that BACP facilitates a more efficient remineralization process. The higher microhardness values correlate with the uniform and dense mineral deposition observed in SEM images. Additionally, the increased Ca/P ratio in BACP-treated samples rejects the null hypothesis that the presence of trace elements enhances its remineralization potential. The integration of these findings aligns with previous studies that emphasize the role of bioactive ACP formulations in promoting dentin repair and preventing demineralization. This study has certain limitations, including the lack of evaluation of the intrafibrillar mineralization capability of BACP and the absence of specific molecular studies to confirm its antibacterial effect. Additionally, the study did not assess long-term ion release or the depth of mineral penetration into dentinal tubules, which are critical for sustained therapeutic effect. Although the antibacterial potential and remineralization efficacy of BACP were demonstrated, future research should focus on exploring its potential for intrafibrillar remineralization and conducting molecular-level studies to establish a more comprehensive understanding of its structural integration within the dentin matrix. Also, the comparative remineralization potential with commercial agents like fluoride and bioactive glass can also be validated to incorporate it into various restorative materials and dentin adhesives.

The findings of this study have important implications for the development of sustainable, multifunctional dental biomaterials. Clinically, BACP could be integrated into desensitizing agents, remineralizing pastes, liners, or bioactive fillers in restorative composites. Its dual action enhancing mineral recovery and reducing bacterial load addresses two core etiological factors in caries progression: demineralization and microbial activity. This could potentially reduce the incidence of secondary caries, improve restoration longevity, and reduce the need for aggressive mechanical interventions. Furthermore, the trace elements naturally present in BACP (e.g., Mg, Sr, Zn) could stimulate odontogenic differentiation and promote tissue repair, indicating its possible future application in regenerative endodontics and dentin-pulp complex engineering. However, transitioning from laboratory to chairside application requires further evaluation under dynamic oral conditions, long-term in vivo studies, and formulation compatibility testing with existing dental materials. Overall, this study provides foundational evidence supporting BACP as a next-generation biomaterial in conservative and preventive dentistry, bridging the gap between sustainability, efficacy, and biological safety.

## Conclusion

Within the limitation of the study, it can be concluded that BACP exhibits superior remineralization potential and antibacterial activity than SACP. Thus, the superior performance of BACP in terms of remineralization, antibacterial efficacy, and biocompatibility suggests that it could serve as a cost-effective and environmentally friendly alternative to synthetic calcium phosphates in restorative dentistry.

## Data Availability

The datasets used and/or analysed during the current study are available from the corresponding author on reasonable request.

## Abbreviations

ACP: Amorphous Calcium Phosphate
SACP: Synthetic Amorphous Calcium Phosphate
BACP: Biogenic Amorphous Calcium Phosphate
SEM: Scanning Electron Microscopy
EDX: Energy-Dispersive X-ray Spectroscopy
FTIR: Fourier-Transform Infrared Spectroscopy
XRD: X-ray Diffraction
MTT: 3-(4,5-dimethylthiazol-2-yl)-2,5-diphenyltetrazolium bromide (a cell viability assay)
hDPSCs: Human Dental Pulp Stem Cells
HAp: Hydroxyapatite
TCP: Tricalcium Phosphate
BCP: Biphasic Calcium Phosphate
CDHA: Calcium Deficient Hydroxyapatite
Ca²⁺: Calcium Ion
PO₄³⁻: Phosphate Ion
Mg: Magnesium
CPP: Casein Phosphopeptides
PAA: Polyacrylic Acid
CaCO₃: Calcium Carbonate
CaO: Calcium Oxide
Ca(OH)₂: Calcium Hydroxide
Ca/P: Calcium to Phosphorus Ratio
DS: Demineralization Solution
AS: Artificial Saliva
SBF: Simulated Body Fluid
ISO/EN 10993-12: International Standard for Biological Evaluation of Medical Devices
RPM: Revolutions Per Minute
VMH: Vickers Microhardness
BMH: Baseline Microhardness
DMH: Demineralized Microhardness
RMH: Remineralized Microhardness
SD: Standard Deviation
SPSS: Statistical Package for the Social Sciences
ANOVA: Analysis of Variance
K-S Test: Kolmogorov-Smirnov Test
ROS: Reactive Oxygen Species
